# Secondary Metabolites from Plants Possessing Inhibitory Properties against Beta-Amyloid Aggregation as Revealed by Thioflavin-T Assay and Correlations with Investigations on Transgenic Mouse Models of Alzheimer’s Disease

**DOI:** 10.3390/biom10060870

**Published:** 2020-06-06

**Authors:** Raluca Stefanescu, Gabriela Dumitriṭa Stanciu, Andrei Luca, Luminita Paduraru, Bogdan-Ionel Tamba

**Affiliations:** 1 Center for Advanced Research and Development in Experimental Medicine (CEMEX), “Grigore T. Popa” University of Medicine and Pharmacy, 700115 Iasi, Romania; raluca.stefanescu@umfiasi.ro (R.S.); andrei.g.luca@umfiasi.ro (A.L.); bogdan.tamba@umfiasi.ro (B.-I.T.); 2 Department of Pneumology, “Grigore T. Popa” University of Medicine and Pharmacy, 700115 Iasi, Romania; 3 Division Neonatology, Department Mother & Child Care, “Grigore T. Popa” University of Medicine and Pharmacy, 700115 Iasi, Romania; luminita.paduraru@gmail.com; 4 Department of Pharmacology, Clinical Pharmacology and Algesiology, “Grigore T. Popa” University of Medicine and Pharmacy, 700115 Iasi, Romania

**Keywords:** Alzheimer’s disease, neurodegenerative disease, beta-amyloid aggregation inhibitors, transgenic mouse model, naturally occurring polyphenol compounds

## Abstract

Alzheimer’s disease is a neurodegenerative disorder for which there is a continuous search of drugs able to reduce or stop the cognitive decline. Beta-amyloid peptides are composed of 40 and 42 amino acids and are considered a major cause of neuronal toxicity. They are prone to aggregation, yielding oligomers and fibrils through the inter-molecular binding between the amino acid sequences (17–42) of multiple amyloid-beta molecules. Additionally, amyloid deposition causes cerebral amyloid angiopathy. The present study aims to identify, in the existing literature, natural plant derived products possessing inhibitory properties against aggregation. The studies searched proved the anti-aggregating effects by the thioflavin T assay and through behavioral, biochemical, and histological analysis carried out upon administration of natural chemical compounds to transgenic mouse models of Alzheimer’s disease. According to our present study results, fifteen secondary metabolites from plants were identified which presented both evidence coming from the thioflavin T assay and transgenic mouse models developing Alzheimer’s disease and six additional metabolites were mentioned due to their inhibitory effects against fibrillogenesis. Among them, epigallocatechin-3-gallate, luteolin, myricetin, and silibinin were proven to lower the aggregation to less than 40%.

## 1. Introduction

Despite decades of preclinical and clinical research, Alzheimer’s disease (AD), a multifactorial neurodegenerative disorder that involves several pathogenetic mechanisms is still a major and increasing challenge in terms of global health [1,2]. Abnormal aggregation of beta-amyloid (Aβ) may be the primary hallmark noticed in pathogenesis of this condition. The cleavage of amyloid precursor proteins (APP) by β- and γ-secretase to the Aβ peptides followed by soluble Aβ monomers aggregate to β-sheet-rich oligomers and insoluble amyloid fibrils yields in the extracellular medium to senile plaques. Current data on amyloid-beta plaque formation, which is characterized by misfolding, aggregation, and deposition of Aβ peptide and leads to cellular dysfunction, loss of synaptic transmission, and brain impairment indicate that AD is one of the protein misfolding disorders (PMDs). A recent step forward in the field was obtained through experimental results indicating that misfolded protein aggregates such as amyloid-beta in AD, human islet amyloid polypeptide in type 2 diabetes, or α-synuclein in Parkinson’s disease could self-disseminate by seeding and spread the pathological deficits between cells and tissues [3,4]. This breakthrough has broad implications for understanding the pathways implicated in the onset and evolution of AD, as well as for the design of new plans and strategies for treatment and diagnosis [5,6,7].

**Figure 1:**
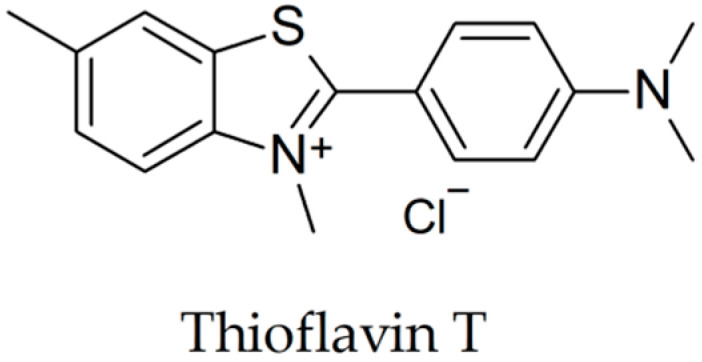
Chemical structure of thioflavin T.

The aggregation of beta-amyloid peptides has been investigated extensively in the last 20 years [8]. In the amyloid fibrils, the amino-terminal end of the amyloid-beta peptide is exposed to the interaction with other molecules, while the middle region and the carboxyl-terminal end of the peptide are involved in intramolecular and intermolecular interactions between molecules of Aβ [9]. Molecules which belong to various classes were analyzed in previous studies as non-covalent binding partners for Aβ peptide: antibodies, peptides, proteins and low molecular weight molecules [3,4,8,10]. The methods employed most often for comparing aggregation, and particularly amyloid fibril formation using different experimental conditions, are the thioflavin T assay (Figure 1), transmission electron microscopy, atomic force microscopy, and scanning electron microscopy. Sandwich ELISA and UV spectroscopy were also described for quantifying the degree of aggregation, although these methods do not distinguish between the form of the aggregated peptide, namely oligomers or fibrils [11,12].

Beta-amyloid fibrils and oligomers bind thioflavin T molecules, while monomers of beta-amyloid do not interact with this chemical compound [13,14]. The excitation spectra of thioflavin T solubilized at a concentration of 3 µM in 50 mM potassium phosphate buffer shows a maximum at 450 nm in the presence of Aβ(1-28) and Aβ(1-40). The signal recorded at 450 nm had low intensity when thioflavin T was measured alone. In the emission spectra, a maximum was recorded at 482 nm for both Aβ(1-28) and Aβ(1-40) incubated with thioflavin T, while thioflavin T alone did not present a signal at this wavelength [15]. Although the molecular basis of the interaction between thioflavin T and amyloid-beta fibrils is not completely understood, it is hypothesized that thioflavin T binds to the β-sheet resulted through the interaction of the sequences (17–42) belonging to a high number of Aβ(1-42) molecules [16].

**Figure 2:**
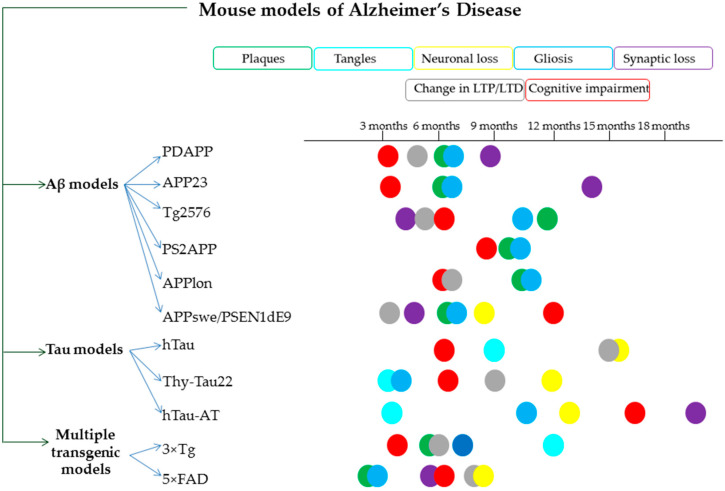
Schematic representation of the most common mouse transgenic models of Alzheimer’s Disease (AD) in association with their pathological changes and neurological deficits. Aβ models: PDAPP (hAPP695, 751 and 770 with Indiana V717F transgene, PDGF promoter), APP23 (hAPP751 containing the Swedish KM670/671NL transgene, Thy-1 promoter), Tg2576 (hAPP695 with Swedish transgene KM670/671NL, HamPrP promoter), PS2APP (hAPP695 with Swedish mutation KM670/671NL and PSEN2 with the N141I Volga German transgenes, Thy-1.2 promoter), APPlon (hAPP695 with Indiana mutation V717I, Thy1 promoter), APPswe/PSEN1dE9 (hAPP695 with Swedish mutation KM670/671NL, PSEN1:deltaE9 transgenes); tau models: hTau (human tau), Thy-Tau22 hTau (transgene containing the cDNA of the 412 amino acid isoform of human 4-repeat Table 272. V and P301S transgene), hTau-AT (hTau40 isoform 2N4R with the A152T transgene), and multiple transgenic models: 3xTg-AD (hAPP695 with Swedish KM670/671NL transgene Thy1 promoter; hTau with P301L, 0N4R mutation, Thy1 promoter;PSEN1 with M146V mutation, PS1promoter; 5xFAD (hAPP695 with Swedish, London and Florida mutations; PSEN1 with M146L and L28V mutations, Thy1 promoter). LTP, long-term potentiation; LTD, long-term depression of excitatory synaptic transmission.

The insufficient therapeutic efficacy of currently approved drugs necessitates the introduction of better in vivo preclinical models able to reproduce AD pathology, especially in the pre-symptomatic phase, and then to explore useful tools for preventive and therapeutic screening [17,18]. In response to this need, more than 180 transgenic/knock-out/knock-in AD models have been developed that are mostly centered alone or in combination with human gene mutations in *APP*, presenilin 1 *(PSEN1*), apolipoprotein E (*APOE*), microtubule-associated protein tau (*MAPT*), and triggering receptor expressed on myeloid cells2 (*Trem2*) or Beta-Secretase 1 (*BACE1*). Figure 2 presents the pathological changes and the neurological deficits of the most common mouse transgenic models of AD used in research: Aβ models: PDAPP [19], APP23 [20,21], Tg2576 [22,23], PS2APP [24], APPlon [25], APPswe/PSEN1dE9 [26]; tau models: hTau [27], Thy-Tau22 [28], hTau-AT [29] and multiple transgenic models: 3×Tg-AD [30] and 5xFAD [31]. Although none of these models completely replicates the most important features of human AD, in vivo models provide context and relevance insight into the pathological alterations that define this condition [32,33,34,35,36].

In a previous study performed by Stefanescu and colleagues [37], the authors searched for amyloid-beta aggregation inhibitors, using as a literature search, criterion the mass spectrometric methods developed for the analysis of non-covalent complexes. In the present study, the authors aimed to find, by searching the scientific literature, natural plant derived products for which there is experimental evidence obtained using thioflavin T assay, indicating an inhibitory activity of these chemical compounds towards amyloid-beta fribrillogenesis. A literature search was performed for all the compounds found, in order to verify whether there are studies carried out using the same natural products on transgenic mouse models developing Alzheimer’s disease. The results from both types of studies are presented in subchapters dedicated to each secondary metabolite from plants.

## 2. Secondary Metabolites from Plants Identified as Inhibitors of Amyloid-Beta Fibrillogenesis

### 2.1. Gallic Acid

Liu et al. investigated the capacity of gallic acid to block the formation of amyloid-beta fibrils using a solution of 50 μM Aβ(1-40) incubated during 10 h and thioflavin T assay. They found that a solution of 100 μM gallic acid prevented the fluorescence produced by thioflavin T molecules when these are found in bound state to the Aβ(1-40) fibrils [38]. Transmission electron microscopy images acquired after 20 h of incubation indicated also that in the presence of two-fold molar excess of gallic acid mature fibrils were not formed [38]. In another study, the fibrillization of Aβ(1-42) was monitored by thioflavin T assay in the absence and in the presence of 100 μM gallic acid. Although the thioflavin T assay results showed a diminished fluorescence of thioflavin T when Aβ(1-42) was incubated with gallic acid, the transmission electron microscopy (TEM) indicated that fibrils were present and gallic acid may interfere with Aβ binding [39]. Porzoor et al. incubated Aβ(1-42) for 24 h in the absence or presence of gallic acid at a final solution concentration of 20 μM amyloid-beta peptide and inhibitor. Thioflavin T exhibited a decrease of the fluorescence in the presence of gallic acid [40]. Yu Mei et al. reported experimental evidence indicating that preformed Aβ(1-42) fibrils at 20 μM concentration treated with two-fold molar excess of gallic acid underwent a disaggregation process [41].

**Figure 3:**
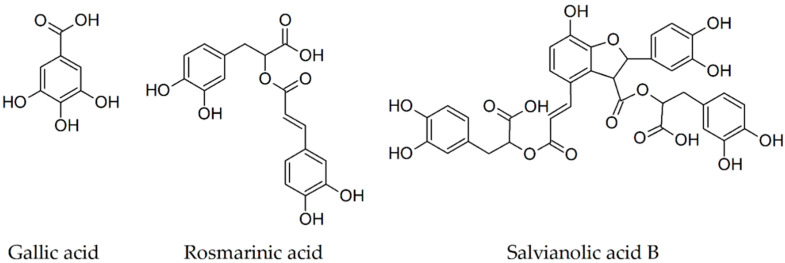
Chemical structures of phenolic acids from plants whose inhibitory activity towards beta-amyloid fibrillogenesis was studied.

The anti-amyloidogenic activity of gallic acid through gavage against cerebral Aβ/β-amyloid pathology at a daily dose of 30 mg/kg (dissolved in sterile water at the concentration of 3 mg/mL) for 30 days in APP/PS1 double transgenic mice (starting at 4 or 9 months of age) was evaluated by Yu et al. [41]. After one month of therapy with gallic acid, the brain sections of the 9-month-old APP/PS1 mice stained either with thioflavin S or with an amyloid plaque specific antibody 6E10 revealed an important reduction in the size of Aβ1-42 plaques, not in their numbers. In addition, GA in a 9-day behavioral assessment battery not only proved to be effective in improving the spatial reference and working memories of 4-month-old transgenic mice (low plaque stage), but also significantly reduced the most severe deficits of cognitive functions developed by the 9-month-old AD mice (high amount of brain Aβ plaque depositions). Taken together, these findings lead to fact that gallic acid (Figure 3) shrank the size of Aβ aggregates, although the mechanism of action remains unclear and possibly prevented plaque development in the brain. All data support the idea that gallic acid may be added in the future as a possible multitarget pharmacological compound in the prevention or treatment of Alzheimer’s disease.

### 2.2. Rosmarinic Acid

A 24 h incubation of Aβ(1-42) and rosmarinic acid at a final concentration of 20 µM and analysis of the resulting solution by thioflavin T assay, led to a very low intensity of thioflavin T fluorescence when compared to the peptide incubated in the vehicle buffer [40]. A similar result was obtained by Sun et al. when analyzing the intensity of thioflavin T fluorescence of a solution of Aβ(1-42) incubated with 1, 10, and 100 µM rosmarinic acid for 24 h [42].

Long-term oral administration of rosmarinic acid (diet containing 0.5% of phenolic compound) for 10 months in transgenic female Tg2576 mice was correlated with an important reduction of tris-buffered saline (TBS)-insoluble beta-amyloid peptides, namely Aβ(1-40) and the total Aβ(1-40) and Aβ(1-42) quantified in the precipitate, resulted after centrifugation of mice brain homogenates and resuspension in 5 M guanidine. Similarly, a reduction of the Aβ oligomers recognized by the A11 antibody, which interacts specifically with oligomers and does not form immune complexes with beta-amyloid monomers or with fibrils, was quantified. A remarkable increase in TBS-soluble beta-amyloid peptides, namely Aβ(1-42) and the total Aβ(1-40) and Aβ(1-42), was quantified in the supernatant fraction. Moreover, the authors reported a decrease in the number of beta-amyloid plaques. These data suggest that rosmarinic acid can inhibit the Aβ aggregation pathway from Aβ monomers to A11-positive oligomers and from A11-positive oligomers to Aβ deposition [43]. To explain the mechanism of suppression of Aβ aggregation by rosmarinic acid, Hase et al. [44] evaluated, using DNA microarray analysis, the brains of transgenic Tg2576 mice fed a diet containing 0.5% rosmarinic acid for 10 months. The results revealed that dopamine secretion and the dopaminergic synapse pathway were enhanced in the group of mice treated with rosmarinic acid. Moreover, the therapy with rosmarinic acid led to an increase of monoamines levels in the cerebral cortex. Through the decrease of Aβ aggregation by increasing in the brain, the concentration of monoamine secretion, dietary supplementation of RA (Figure 3) may positively act in prevention and treatment of Alzheimer’s disease.

### 2.3. Salvianolic Acid B

According to experimental data published by Durairajan et al., salvianolic acid B inhibits Aβ(1-40) fibril formation in a dose-dependent manner, having maximum inhibitory effect at 100 μM. Moreover, the addition of an increasing amount of salvianolic acid B to preformed fibrils obtained by four-days incubation of a Aβ(1-40) solution containing 15 μM peptide results in a dose-dependent reduction of thioflavin T fluorescence [45]. In transmission electron microscopy, the same researchers observed that addition of 1 μM salvianolic acid B solution at the beginning of the aggregation or after four-days preincubation of a 50 μM solution, Aβ(1-40) led to the formation of short fibrils and amorphous structures of Aβ(1-40). Porzoor et al. incubated Aβ(1-42) for 24 h in the absence or presence of salvianolic acid B at a final solution concentration of 20 μM amyloid-beta peptide and inhibitor and observed a lowered fluorescence intensity in the presence of salvianolic acid B [40].

The data reported by Shen et al. [46] regarding daily intraperitoneal treatment with total salvianolic acid at doses of 30 and 60 mg/kg for 14 weeks in APPswe/PS1dE9 mice revealed not only a reduction of spatial cognitive impairments through decreasing Aβ(1-42) and Aβ(1-40) levels, but also an improvement of different other metabolic markers. This includes a decreased plasma low-density lipoprotein cholesterol level, which seems to be positively associated in the hippocampus with Aβ(1-42) levels. The results of this study suggested that total salvianolic acid included a multi-metabolite regulator whose mechanism was involved in decreasing the amount of Aβ(1-40) and Aβ(1-42) by inhibiting the plasma low-density lipoprotein cholesterol level, the production or the activity of the secretase involved in the amyloidogenic pathway. In AD management, the salvianolic acid (Figure 3) can be a promising therapeutic agent due to its long-term protective effects on learning and memory by regulating metabolites.

### 2.4. Luteolin

In a study published in 2008, Akaishi et al. demonstrated experimentally using thioflavin T assay and the peptide Aβ(1-42), that 100 μM luteolin was able to decrease the intensity of thioflavin T fluorescence to a very low value when compared with the peptide measured in the vehicle [47]. The investigation by thioflavin T assay of the extent of Aβ(1-42) fibrillization at the concentration of 40 μM for both peptide and luteolin resulted in a strongly reduced fluorescence intensity of thioflavin T [48].

**Figure 4:**
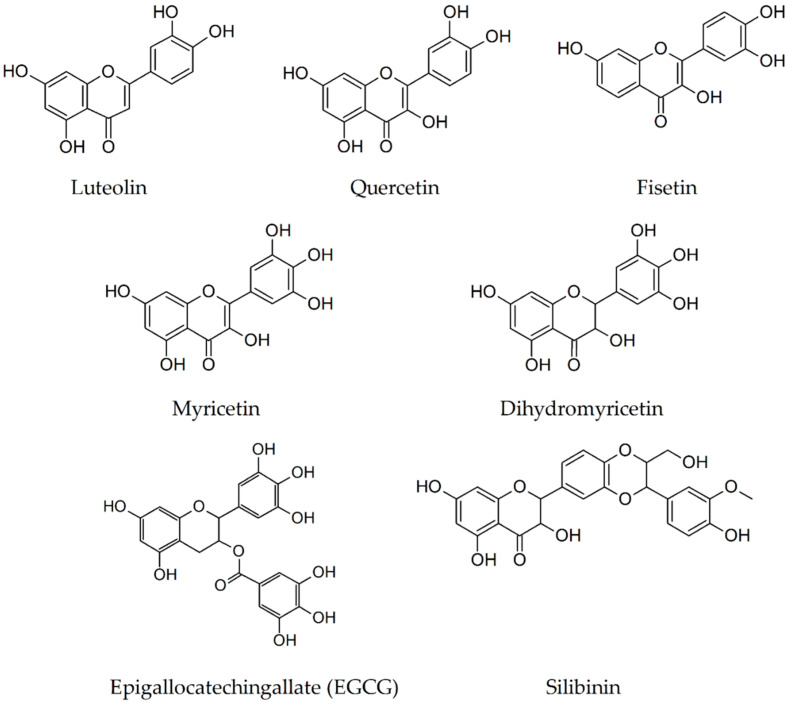
Chemical structures of flavonoids and flavanol-lignan silibinin from plants whose inhibitory activity towards beta-amyloid fibrillogenesis was studied.

Preclinical research using transgenic mouse models of AD revealed that luteolin (Figure 4) is able to reduce amyloidogenesis determined by APP mutations related with familial AD [49] and traumatic brain injury [50]. Intraperitoneal treatment for 30 days with luteolin (20 mg/kg/day) in elderly Tg2576 mice significantly attenuated the cognitive impairments and ELISA assay revealed an inhibition of soluble Aβ(1–40) and Aβ(1-42) generation by 25% and 49%, respectively. The mechanism behind the decrease of brain Aβ accumulation is most likely represented by a selective inactivation of glycogen synthase kinase 3α vital for both PSEN1 processing/phosphorylation and interaction between APP and PSEN1 [49].

### 2.5. Quercetin

The inhibitory activity of quercetin on Aβ(1-42) fibril formation was also analyzed by thioflavin T assay [47]. Quercetin was incubated at three concentrations 1, 10, and 100 μM with 20 μM of Aβ(1-42) in vehicle and the values recorded for the thioflavin T fluorescence intensity were compared with the values recorded for the sample containing Aβ(1-42) in vehicle. According to the results, a diminished fluorescence intensity was obtained for the sample containing 100 μM quercetin.

The effects of 12 months gavage quercetin therapy on neurodegeneration markers, cognitive and emotional impairments in a triple transgenic (3xTg-AD) mouse model of AD using histological and behavioral analyses were evaluated by Perez-Corredor et al. [51]. Long-term treatment with 100 mg/kg quercetin, every 48 h, administered orally, has substantial effects on β-amyloidosis decrease, and in the hippocampus and amygdala, it tends to reduce tau pathology. These findings positively impacted the cognitive functional recovery without altering the emotional abilities of these transgenic mice. These data are in agreement with a previous study that demonstrated that chronic treatment with quercetin for 3 months (intraperitoneal injection of 25 mg/kg/48 h quercetin dissolved in PBS containing 0.1% DMSO) on aged 3xTg-AD mice reduced tauopathy and extracellular amyloidosis protecting the emotional and cognitive functions [52].

Zhang et al. [53] found that a daily dose of 500 mg/kg of quercetin suspended in corn oil, orally administered for 10 consecutive days on transgenic 5xFAD mice, increases in the cortex the levels of apolipoprotein E fragments (apoE3 and apoE4) and decreases insoluble Aβ(1-40) levels evaluated by Western blot and real-time PCR. Increased levels of apoE lead to the clearance of Aβ(1-42), and may reverse memory deficits in AD mouse models. As a conclusion of these results, it is clear that the quercetin (Figure 4) therapy might delay the development and progression of histopathological hallmarks and cognitive decline in AD.

### 2.6. Fisetin

A solution containing final concentrations of 20 μM Aβ(1-42) and 100 μM fisetin was incubated for eight hours at 37 °C. When using the thioflavin T assay, the intensity of the ThT fluorescence was reduced to approximately 60% of the value obtained by incubating the peptide Aβ(1-42) with the vehicle (0.2% dimethyl sulfoxide) [47].

The effects on AD transgenic mice exerted by fisetin, a flavonol compound that in vivo possesses multiple well-known neuroprotective properties and additionally neurotrophic and anti-amyloid activities in vitro, was investigated by Currais et al. [54]. A daily oral dose of approximately 25 mg/kg (0.5% compound in the diet) of fisetin administered from 3 to 12 months of age prevented progressive memory loss and learning impairments. The compound (Figure 4), however, did not modify the formation of amyloid plaques, deposits of proteins that are frequently associated with Alzheimer’s disease. The results propose a way to treat AD signs individually on targeting amyloid plaques.

### 2.7. Myricetin

A solution containing final concentration of Aβ(1-42) of 10 µM and variable final concentrations of myricetin (0.1, 0.3, 1, 3, and 10 µM) was incubated at 37 °C for 48 h. At the concentrations of 3 and 10 µM, the results of the thioflavin T assay indicate that the fluorescence decreased to approximately 50% [55]. Akaishi et al. obtained a reduction of the fluorescence to approximately 20% of the value obtained for 20 µM Aβ(1-42) and vehicle using final concentration of 20 µM Aβ(1-42) and 10 or 100 µM myricetin [47].

Long-term oral myricetin therapy (including 0.5% phenolic compound in the diet for 10 months) in Tg2576 AD females from 5 months of age was used by Hamaguchi et al. [43] to study different aspects of the Aβ aggregation pathway. The treatment was associated with a reduction in A11-positive oligomers and a tendency to attenuate Aβ plaque deposition without reaching a statistically significant level. The drug does not influence Aβ fibrillization. These results support the idea that myricetin (Figure 4) can inhibit the Aβ aggregation pathway from Aβ monomers to A11-positive oligomers, accelerating the pathway from A11-positive oligomers to Aβ deposition. Therefore, myricetin could be an attractive therapeutic candidate for preventing AD because it inhibits Aβ oligomerization.

### 2.8. Dihydromyricetin

The aggregation of the peptide Aβ(1-40) solubilized in 100 mM PBS, 10 mM NaCl, pH 7.4 at the final concentration of 30 μM was monitored by thioflavin T assay in the absence and presence of 1, 3, 10, 30, and 90 μM dihydromyricetin. The intensity of the fluorescence decreased to less than 25% for the sample containing Aβ(1-40), incubated with 90 μM dihydromyricetin considering the fluorescence intensity of Aβ(1-40) as 100%. An additional assay was carried out for the investigation of the effect of dihydromyricetin on preformed fibrils obtained by incubating Aβ(1-40) for 7 days, followed by incubation for 72 h with dihydromyricetin, and observed a reduction in ThT fluorescence intensity. The analysis by AFM of a sample containing 30 μM Aβ(1-40) incubated for 120 h indicates formation of amyloid fibrils, while analyzing a sample containing 30 μM Aβ(1-40) and 90 μM dihydromyricetin led to the observation of amorphous aggregates. In agreement with the ThT results, the AFM analysis of a sample containing preformed Aβ(1-40) fibrils incubated with 90 μM dihydromyricetin for an additional 72 h indicated that the sample did not contain fibrils [56].

Chronic oral administration of dihydromyricetin (2 mg/kg/day in 2% sucrose) in two different transgenic mouse models of AD (TG2576 and TG-SwDI) has been correlated with a significant amelioration of behavioral deficits and with a reduction in learning and cognitive impairments. Moreover, dihydromyricetin (Figure 4) therapy for 3 months reduced the pathological accumulation of both Aβ1–40 and Aβ1–42 in the brain of TG-SwDI mice by restoring gephyrin (a postsynaptic gamma-aminobutyric acid protein –GABA, that regulates the formation and plasticity of GABAergic synapses) to control levels, GABA transmission, and functional synapses [57].

### 2.9. Epigallocatechin-3-Gallate

Churches et al. obtained a decrease to less than 20% of the fibrillization process for Aβ(1-42) when the peptide was incubated with epigallocatechin-3-gallate (EGCG), both at a final concentration of 40 μM [41]. A similar result was obtained when epigallocatechin-3-gallate was incubated with Aβ(1-42) at 20 μM and 1:1 molar ratio [33]. Huang et al. obtained a complex through the combination of EGCG and the peptide sequence KLVFF, the later representing the sequence [16,17,18,19,20] of the peptide Aβ(1-40). The analysis of the Aβ(1-42) fibrillogenesis by thioflavin T assay led to the conclusion that when Aβ(1-42) and KLVFF/EGCG complex were incubated, the intensity of thioflavin T fluorescence was very low [58]. Reduction and loss of ThT fluorescence was obtained when fibrils of Aβ(1-42) were incubated with EGCG at 1:1 and 1:5 ratios, respectively [59].

In the last decades, the neuroprotective properties of epigallocatechin-3-gallate (EGCG) in the prevention and therapy of AD have been verified on different AD mice models. In transgenic APPswTg 2576 mice, intraperitoneal treatment with 20 mg/kg/day of EGCG for 2 months was associated with a substantial reduction in cerebral Aβ levels; soluble Aβ(1–40) and Aβ(1-42) levels were decreased by ~54 and 44%, respectively; while insoluble Aβ(1–40) and Aβ(1-42) by 47 and 38%, respectively. In addition, immunohistochemistry and thioflavin S histochemistry analyses revealed a considerable decrease of amyloid plaques formation by 47–54% in hippocampal region and by 35–46% in cortical brain area. These evidences are associated with an amplified generation of nonamyloidogenic APP fragments (α-CTF and sAPP-α) and a prominent α-secretase cleavage activity by about 40%, suggesting that EGCG supports nonamyloidogenic processing of APP and diminishes cerebral amyloidosis [60]. Using the same transgenic mouse strain, Rezai-Zadeh and colleagues [61], in a subsequent study investigating the effects of oral administration of EGCG (50 mg/kg/day for 6 months in drinking water) on cognition, Aβ aggregation, and tau pathology, reported similar results. Moreover, the treatment exhibited beneficial cognitive effects in radial arm water maze evaluation and led to a reduction in the toxic potential of soluble sarcosyl phospho-tau isoforms. These data were in agreement with those reported by Li et al. [62] who explored the ability of EGCG (oral gavage, 20 mg/kg/day for 3 months) to interfere with Aβ plaque deposits in various areas of the brain. The EGCG consumption was able to reduce the Aβ deposits by 60% in the frontal cortex region and by about 52% in the hippocampus of APP transgenic mice. In another study centered on APP/PS1 (APPswe, PSEN1dE9) double transgenic mice, Jia et al. [63] investigated the effects of long-term gavage of 2 or 6 mg/kg/day of EGCG (dissolved in 0.15 mL saline) for 4 weeks on cognitive functions, Aβ levels, and capacity to inactivate the TNF-a/ JNK signaling pathway to attenuate insulin resistance. The behavioral tests revealed that EGCG ameliorates the spatial learning and memory impairments. Moreover, ELISA and immunohistochemistry evaluations described a consistent decrease of the IRS-1pS636 level, accompanied by a reduction of both soluble and insoluble Aβ(1-42) levels in the hippocampus, in a dose dependent manner. These results support the idea that systemic delivery of EGCG may attenuate brain insulin resistance in animal models of AD. Chronic dietary supplementation with daily 50 mg/kg of EGCG for 4 months, accompanied by a homecage access to a running wheel, was associated with improved spatial learning and nest building skills and decrease of soluble Aβ(1-42) levels by about 25–35% in the cortex and hippocampus of TgCRND8 mice. These data were consistent with an earlier study that used this dose of EGCG in the same strain of mice [61] as well as research using long-term voluntary exercise in transgenic AD mice [64]. Taken together, these findings underline the possibility that dietary EGCG (Figure 4) may offer safe and effective prophylaxis for AD.

### 2.10. Silibinin

Yin et al. reported, in the year 2011, the results of the thioflavin T assay employed for the study of the inhibition of Aβ(1-42) peptide aggregation exerted by increasing concentrations of silibinin (0.1, 1, 10, and 100 μM) incubated with Aβ(1-42) at 20 μM concentration [65]. According to these results, a solution of 100 μM silibinin decreased with 70% the aggregation of the Aβ(1-42). Silibinin, as a dual potential inhibitor of acetylcholinesterase and Aβ peptide aggregation for AD therapy, was evaluated on APPswe/PS1dE9 double Tg mice model by Duan et al. [66]. Intraperitoneal daily administration of 200, 20, or 2 mg·Kg^−1^ of silibinin (suspended in 0.5% carboxymethylcellulose sodium solution) for 4 weeks resulted in an amelioration of cognitive deficits, a remarkable reduction in the surface area of Aβ plaque aggregates in the cortex and hippocampus (mainly 20 mg group), a decrease in the activity and quantity of acetylcholinesterase, as well as an increase in synaptic protection, gliogenesis, and neurogenesis. These data highlight that silibinin (Figure 4) plays an effective role in preventing the aggregation of Aβ by binding to the Aβ1-42, acting not only as a dual inhibitor of Aβ aggregation and acetylcholinesterase, but also as a neurogenic agent, being a “one molecule-multiple targets” promising compound in AD therapy.

### 2.11. Oleuropein

Thioflavin T assay was carried out for the analysis of the inhibitory activity of oleuropein and olive leaves extract towards Aβ(1-42) fibrillogenesis. The results indicated a 61% and 60% inhibition of Aβ(1-42) fibrillogenesis for oleuropein and olive leaves extract respectively.

**Figure 5:**
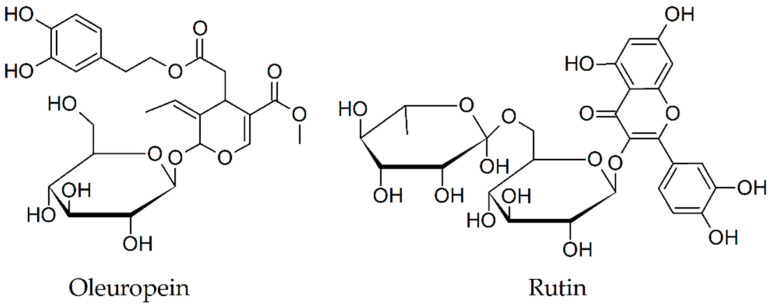
Chemical structures of oleuropein and rutin.

Omar et al. [67] studied the effects of 4-months dietary supplementation of oleuropein (Figure 5) comprising olive leaf extracts (50 mg/kg/day) on amyloid pathology along with possible behavioral alterations in the APPswe/PS1dE9 and wildtype of mice. The therapy revealed a remarkable amelioration of hippocampal neuropathology, leading to a marked reduction of amyloid plaques areas in the cortex and hippocampus of APPswe/PS1dE9 mice compared to the control group. In a previous study on an oleuropein aglycone diet fed (50 mg/kg/day for 8 weeks) in a mice model of amyloid-β deposition (TgCRND8), similar findings have been reported by Grossi et al. [68]. As a mechanism of inhibition of Aβ aggregation by olive biophenols, the authors propose a breakdown of the fibrils formed and the interference with the rates of colloidal aggregation properties and the conformational preference of Aβ, which leads to further inhibition of aggregation.

### 2.12. Rutin

Aβ(1-42) was incubated at a final concentration of 10 μM, alone or with 50 or 200 μM rutin [69]. The samples were analyzed during 24 h, at time intervals of 6 h, using thioflavin T assay. The results indicated a significant decrease of the fluorescence intensity in the thioflavin T assay for the sample containing 50 μM rutin and a very low signal of thioflavin T for the sample containing 200 μM rutin.

In vivo neuroprotective effects of a daily dose of 100 mg/kg rutin orally administrated for 6 weeks were investigated by Xu et al. [70] in a double transgenic APPswe/PS1dE9 mice model. Compared with the control group, the rutin-treated APPswe/PS1dE9 mice showed a reduction in Aβ oligomers levels by approximatively 60.8% and 31.7%, respectively, as identified with oligomer-specific A11 and W20 antibodies in dot-blot analysis correlated with an attenuation of spatial memory deficits. In addition, rutin therapy downregulated microgliosis and astrocytosis; increased the activity of superoxide dis-mutase and antioxidant glutathione and its oxidized form ratio; reduced glutathione peroxidase and malondialdehyde levels, as well as decreased interleukin-6 and interleukin-1β levels in the AD mouse brains. Taken together, these findings suggest that rutin (Figure 5) neuroprotective effects occur by inhibiting the activation of glial cells and attenuating inflammatory cytokine production.

Interestingly, the intravenous delivery of Congo red/rutin magnetic nanoparticles to APPswe/PS1dE9 transgenic mice has been associated with detection of numerous amyloid plaques in enhanced magnetic resonance imaging analysis and with marked improvement in spatial memory. Immunohistochemistry assay and Nissl staining revealed an important reduction in Aβ plaque loads in the AD mouse brains, showing that treatment with Congo red/rutin-magnetic nanoparticles had the capability to decrease Aβ deposits [71].

### 2.13. Curcumin

Jiang et al. [72] studied the process of fibrilization underwent by Aβ(1-42) solubilized at a final concentration of 40 μM and showed that the thioflavin T fluorescence intensity increases when Aβ(1-42) is mixed with 10 μM Al(III) and strongly decreases in the presence of 10 μM curcumin even if the Aβ(1-42) was preincubated for three days with 10 μM Al(III).

Systemic curcumin treatment of APPswe/PS1dE9 mice aged 7.5–8.5 months for 7 days (7.5 mg/kg/day in phosphate-buffered saline, delivered intravenously via tail vein) was able to clear and reduce existing plaque deposits (~30%), suggesting an ability of curcumin to disaggregate and inhibit Aβ aggregation. In vivo multiphoton microscopy evaluation revealed that curcumin penetrates the blood-brain barrier, labels plaque deposits and brain amyloid angiopathy. Curcumin also resulted in a limited but substantial reversal of the structural changes of dystrophic dendrites, comprising an altered curvature and size of dystrophy. These data support the idea that curcumin may reverse existing amyloid plaque deposits and associated neurotoxicity [73]. Studying the efficacy of the potential properties of curcumin to inhibit Aβ aggregation in transgenic mice models of AD, Yang et al. [12] revealed that brain sections of the APPsw Tg2576 mice incubated with this compound highlighted preferential labeling of amyloid plaques. Administered peripherally to aged APPsw Tg2576 mice (until 22 months), curcumin crossed the blood-brain barrier and bound plaques. Chronic therapy for 5 months with low doses of curcumin formulated in chow (500 ppm) led to a reduction of Aβ levels by 40% and produced a 43% decrease in Aβ deposits compared with control group. Lim and colleagues [74] tested on Tg2576 mice the effects of dietary doses of curcumin (a low dose—160 ppm and a high dose—5000 ppm of compound) on plaque pathology, oxidative damage and inflammation. The drug considerably reduced oxidized proteins and interleukin-1beta, an increased proinflammatory cytokine in the brains of these mice. The curcumin therapy with low doses reduced the astrocytic marker GFAP and decreases by 43–50% plaque burden, insoluble and soluble Aβ. It was found that in combating the neurodegenerative process in AD, curcumin in low doses administered over a longer period were more effective than high doses. At a higher concentration, curcumin binds to Aβ and blocks it’s self-assembly.

**Figure 6:**
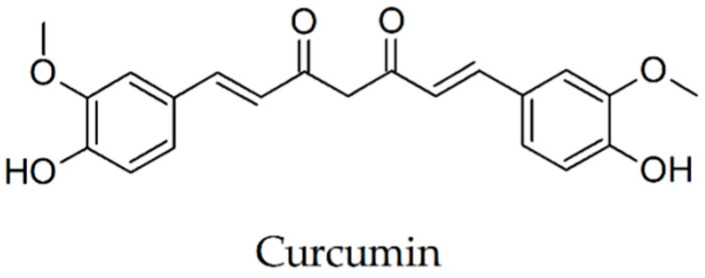
Chemical structure of curcumin.

Wang et al. [75] in a research on APPswe/PS1dE9 transgenic mice evaluated the efficacy and mechanisms of curcumin gavage on the prevention and therapy of AD in doses of 400, 200 and 100 mg/kg/day (suspended in 0.5% sodium carboxymethyl cellulose solution) for 3 months. Behavioural tests have shown that medium- and high-doses of curcumin treatment can improve spatial learning and memory capacity in these mice. Immunohistochemical and Western blot analyses revealed a reduction of Aβ(1-40), Aβ(1-42) and aggregation of amyloid-β in the CA1 hippocampal area; a decrease in the expression of the γ-secretase component presenilin-2 and an intensification in the expression of amyloid-β degrading enzymes. These results suggest that curcumin may reduce pathological aggregation of Aβ, perhaps through mechanisms that inhibit its production, inhibiting presenilin-2 or increasing its clearance by growing degrading enzymes. Due to its multi-target effects, curcumin (Figure 6) is one of the most effective and interesting agents for the development of AD therapeutics.

### 2.14. Crocin

The fluorescence intensity recorded for Aβ(1-40) (0.23 mM) and Aβ(1-42) (0.22 mM) after 2 and 3 h respectively of incubation at 37 °C decreased to 63% and 66% in the presence of crocin at the concentration of 15.4 μM when compared with the fluorescence intensity of the amyloid peptides incubated alone [76,77].

**Figure 7:**
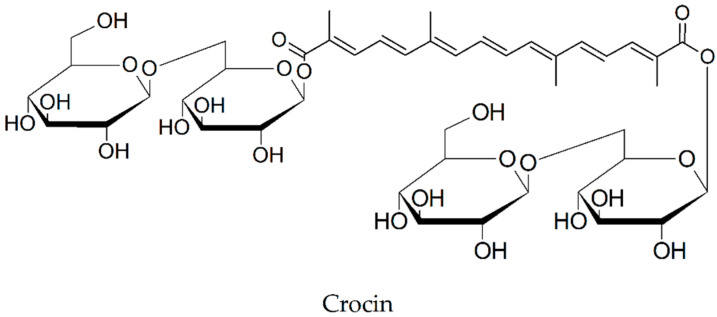
Chemical structure of crocin.

The effects of 1-month dietary supplementation of crocin (10 mg/kg/day) on beta-amyloid load and related toxicity were evaluated in 5XFAD transgenic and wild-type mice by Batarseh et al. [78]. Using ELISA, the researchers showed that crocin (Figure 7) consumption was able to decrease Aβ(1-40) by 25% and Aβ(1-42) levels by 29% in brain homogenates compared with control group. At least in part, the reduction in total Aβ levels might be explained by an increase in the expression of neprilysin (NEP) and up-regulation of the ApoE-clearance pathway.

### 2.15. Cryptotanshinone

The effect of cryptotanshinone on the Aβ(1-42) fibril formation was investigated by Mei and colleagues using thioflavin T assay [79]. The formation of fibrils was analyzed in the absence and presence of 1, 2.5, and 5 μM cryptotanshinione after 72 h incubation at 37 °C of the peptide Aβ(1-42) at 10 μM concentration. The intensity of the fluorescence decreased with the addition of increasing concentrations of cryptotanshinione. In the case of the sample containing 5 μM cryptotanshinione, there was a decrease to 44% of the fluorescence intensity recorded for 10 μM Aβ(1-42) incubated alone.

**Figure 8:**
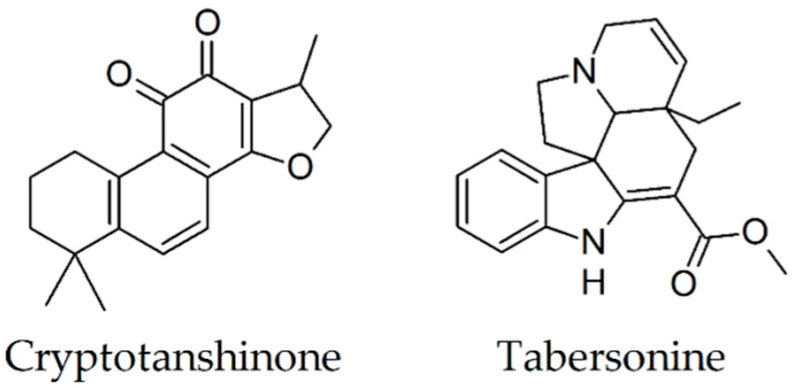
Chemical structures of cryptotanshinone and tabersonine.

Chronic therapy for 4 months with oral cryptotanshinone at the doses of 5, 15, and 30 mg/kg/day was associated in Morris water maze evaluation with an important amelioration of spatial learning and memory deficits of APP/PS1 mice. There was no statistically significant difference between 15 and 30 mg cryptotanshinione-treated groups. In addition, a modified immunohistochemical method revealed significant attenuation of Aβ deposits in the cortex and hippocampus. The authors suggested as the mechanism involved in the positive effects of cryptotanshinone (Figure 8) on β-amyloid deposition attenuation in transgenic AD mice amyloid precursor protein metabolism modulation by upregulating α-secretase [80].

### 2.16. Tabersonine

Thioflavin T assay was employed by Kai et al. for monitoring the formation of Aβ(1-42) fibrils [81]. The results indicated that Aβ(1-42) at 80 μM solubilized in 100 mM phosphate buffer and 100 mM NaCl (pH 7.4) exhibits an increasing intensity of fluorescence in the first 24 h, while the fluorescence intensity remains constant in the following 96 h. At the addition of 10 μM before the beginning of the Aβ(1-42) aggregation process, the intensity of the fluorescence increases for 10 h, exceeding half of the plateau intensity, and decreases in the following 100 h. In a third experiment, 10 μM of tabersonine (Figure 8) were added to preformed fibrils obtained by incubation of Aβ(1-42) for 24 h. The intensity of the fluorescence decreased, indicating a disaggregation process. Atomic force microscopy experiments performed by the same researchers agreed with the results provided by the thioflavin T assay.

The results of the search in the scientific literature are schematically presented in Table 1.

### 2.17. Other Plant Secondary Metabolites

Additional secondary metabolites which reduce the formation of amyloid-beta fibrils to ~20% are maritimetin, robinetin, apigeninidin, and transilitin [48] and cyanidin glucoside [39]. The authors of this review did not find studies using transgenic mouse models developing AD to which one of the latter five plant secondary metabolites were administered as drugs.

## 3. Conclusions

Considering the fact that through the cleavage of amyloid precursor protein isoform 695 existing mainly in the membranes of the neurons by beta- and gamma-secretases and by the cleavage of the isoform 770 of amyloid precursor protein existing mainly in other tissues of the human body a soluble form of amyloid beta peptide results, the authors propose a mechanism in which the secondary metabolites could bind the soluble form of Aβ in blood and could even cross blood–brain barrier and bind soluble Aβ peptides in the CNS reducing their aggregation. An increase in the solubility and excretion of Aβ peptides through the binding of the natural product is desired. According to the results discussed in this review, thioflavin T assay was employed in numerous studies for testing the inhibitory effects of secondary metabolites from plants. In the present study, only the plant secondary metabolites able to diminish the thioflavin T fluorescence to 60% or less than 60% of the value obtained for Aβ(1-40) or Aβ(1-42) incubated in the vehicle were presented. The concentration of amyloid-beta peptides varied in these studies from 10 to 50 μM with two exceptions at tabersonine and crocin where 80 and 230 μM, respectively, where employed. The concentration of inhibitor tested varied from 0.1 to 100 µM. In most of the studies, a concentration of 100 µM of plant secondary metabolite was necessary for a decrease to less than 60%.

The concentrations at which these chemical compounds possess inhibitory properties as revealed by thioflavin T assay are comparable with the concentrations which were employed in mass spectrometric analyses for the observation of non-covalent complexes between amyloid-beta peptides and inhibitors, as reported for the secondary plant metabolite oleuropein and also for melatonin and peptide ligands, namely between 20–50 μM [37]. Further research could be carried out employing affinity chromatography mass spectrometry [88] or direct mass spectrometric analysis of intact noncovalent complexes, both methods having the advantage of the possibility of being coupled with specific and non-specific enzymatic proteolysis of amyloid-beta peptide [89,90]. These studies would offer information on the existence of a non-covalent complex between amyloid beta peptides and the plant secondary metabolites presented in this study and could provide details on the amyloid-beta sequence interacting with the inhibitor of the fibrillogenesis, contributing to the elucidation of the mechanism of action of the beta-amyloid fibrillogenesis inhibitor.

For avoiding the false positive results which may occur in the verification of potential aggregation inhibitors using thioflavin T assay, the process of beta-amyloid fibril formation has to be carefully analyzed in the absence and presence of the substances tested as inhibitors and the fluorescent properties of each inhibitor must be investigated [91,92,93].

The present study underlines the importance of these secondary metabolites in the search for an effective drug against Alzheimer’s disease. Moreover, a future study involving the secondary plant metabolites presented herein, administered separately to the same mouse model of Alzheimer’s disease, could bring further information regarding their molecular mechanisms of action in vivo. These studies should be associated with mass spectrometric determination of drug and of drug metabolites amount in mouse body fluids and brain.

In vivo preclinical models have a crucial significance in understanding the mechanisms of AD, and since, new findings occur in parallel with clinical medicine developments, secondary metabolites from plants identified as inhibitors of Aβ aggregation in this article can be effective agents for the development of AD therapeutics due to their ability to target multiple disease features such as symptomatic therapies, risk factors, or mechanism-based versus non-mechanism based approaches.

Both in vitro and in vivo studies of secondary metabolites from plants presented in this paper confirmed that they have a great influence on the delay and treatment of Alzheimer’s disease. However, the results obtained using preclinical models may not be easy to translate and generalize to humans. In recent decades, an increasing number of studies using polyphenolic compounds, found mainly in red wine, grapes, red fruits, coffee, or green tea [94,95] have focused on their neuroprotective effects in neurodegenerative conditions, as is the case of the recently described neuroprotection against Aβ-mediated neurotoxicity by EGCG [96]. In addition, these research studies also revealed the ability of EGCG to modulate mitochondrial functions [97], mediate autophagy flux [98], cross the human BBB model, and protect cortical cultured neurons from oxidative stress [99]. It is generally known and accepted that most polyphenols are great antioxidants and may also have anti-inflammatory properties. Recent data extended on the initial antioxidant-based mechanism of polyphenols’ activity by displaying that they are also able to modulate numerous cell-signaling pathways and mediators. These properties stimulated the researchers’ interest in polyphenolic compounds and many studies point out their potential role in preventing and treating a wide range of human pathological conditions related to inflammation and oxidative stress, such as cancer, neurodegenerative and cardiovascular disorders [100,101,102]. In clinical trials, curcumin and EGCG confirmed to target Aβ, tau, and transthyretin. Investigating the side effects, drug absorption, and biological effects of curcumin in the treatment of AD patients, Ringman et al. [103] and Baum et al. [104] reported that this molecule had no significant beneficial effects nor serious side events, being able to delay decline, rather than to improve cognitive functions. Interestingly, in the brain, curcumin levels were undetectable, suggesting limited bioavailability. In sum, result studies suggested that the inclusion of polyphenols in the diet or their use as pharmacological drugs, nutraceuticals, or supplements, seems to be promising in the prevention of pathologies with a neurodegenerative nature.

For preventing Alzheimer’s disease, the medicinal plants containing these inhibitors of Aβ fibrillogenesis may be recommended as a healthy diet to young people. In parallel, synthesis of pharmacologically active compounds, followed by preclinical research and clinical trials may lead to complete characterization of the mechanism of action and efficacy of each of these compounds. The bindability and drugability of Aβ(1-40) and Aβ(1-42), which represent also drug target molecules as well as the drug likeness of the secondary metabolites, should be studied further both in reviews gathering data on the current achievement level and through new experiments related to the solubility, absorption, distribution, metabolization and excretion (ADME), structural characterization of the binding sites of the target, determination of the binding affinity [105,106].

## Figures and Tables

**Table 1:** Secondary metabolites from plants possessing inhibitory properties against beta-amyloid aggregation: plant family, in vitro, and in vivo effects.

Secondary Metabolite	Scientific Name of the Plant (Family)	Effects Observed Using Thioflavin T Assay	In Vivo Findings
Gallic acid [38,41]	*Vitis vinifera* (Vitaceae)	diminishes/blocks fibril formation disaggregates preformed fibrils	reduction of Aβ(1-42) plaques size, improvement of the spatial reference and working memories of 4-month-old transgenic mice; reduction of cognitive deficits in 9-month-old AD mice
Rosmarinic acid [40,42,43,44]	*Rosmarinus officinalis* (Lamiaceae)	diminishes/blocks fibril formation in dose-dependent manner	a significant reduction of Aβ plaque number memory improvement increase of TBS-soluble Aβ monomers and reduction of A11-positive oligomers
Salvianolic acid B [45,82]	*Salvia miltiorrhiza* (Lamiaceae)	diminishes fibril formation in dose-dependent manner	decrease of Aβ(1-42) and Aβ(1-40) levels in the hippocampus reduction of spatial cognitive impairments
Luteolin [47,48,60]	*Daucus carota* (Apiaceae)	diminishes fibril formation	inhibition of soluble Aβ(1–40) and Aβ(1-42) generation by 25% and 49%, respectively, attenuation of the cognitive impairments
Quercetin [47,52,53,83,84]	*Malus domestica* (Rosaceae)	diminishes fibril formation	reduction of tauopathy and extracellular amyloidosis
Fisetin [47,54]	*Fragaria moschata* (Rosaceae)	diminishes fibril formation	prevention of progressive memory loss and learning impairments
Myricetin [43,47,55]	*Vitis vinifera* (Vitaceae)	diminishes fibril formation in dose-dependent manner	reduction of the A11-positive oligomers and a tendency to attenuate Aβ plaque deposition
Dihydromyricetin [56,57,85]	*Ampelopsis grossedentata* (Vitaceae)	diminishes/blocks fibril formation in dose-dependent manner, disaggregates preformed fibrils in dose-dependent manner	reduction of Aβ(1-42) and Aβ(1-40) levels, amelioration of behavioral deficits, reduction of learning and cognitive impairments
EGCG [39,48,49,59,62,63,86]	*Thea sinensis* (Theaceae)	diminishes/blocks fibril formation in dose-dependent manner disaggregates preformed fibrils	reduction of the plaque formation, decrease soluble and insoluble Aβ(1-40) and Aβ(1-42), improvement of working memory
Silibinin [65,66]	*Silybum marianum* (Asteraceae)	diminishes fibril formation in dose-dependent manner	a remarkable reduction in the surface area of Aβ plaque, a decrease in the activity and quantity of acetylcholinesterase, and an increase in synaptic protection, gliogenesis, and neurogenesis amelioration of cognitive deficits
Oleuropein [67,68]	*Olea europea* (Oleaceae)	diminishes fibril formation in dose-dependent manner	reduction of Aβ levels and plaque areas in the cortex and hippocampus
Rutin [69,70,71]	*Malus domestica* (Rosaceae)	diminishes/blocks fibril formation in dose-dependent manner	a reduction in Aβ oligomers levels, attenuation of memory deficits, reduction of microgliosis, astrocytosis, glutathione peroxidase, malondialdehyde, interleukin-6, and interleukin-1β levels; increase of glutathione/glutathione disulfide ratio
Curcumin [12,72,74]	*Curcuma longa* (Zingiberaceae)	diminishes/blocks fibril formation in dose-dependent manner, disaggregates preformed fibrils	reduction of soluble and insoluble Aβ, plaque burden, and the astrocytic marker GFAP using low-dose TBS-soluble, reduction of A11-positive oligomers
Crocin [76,77,78]	*Crocus sativus* (Iridaceae)	diminishes fibril formation	decrease of Aβ(1-40) by 25% and Aβ(1-42) levels by 29%, respectively
Cryptotanshinone [79]	*Salvia miltiorrhiza* (Lamiaceae)	diminishes fibril formation	attenuation of Aβ deposits amelioration of spatial learning and memory deficits
Tabersonine [81,87]	*Voacanga africana* (Apocynaceae)	diminishes fibril formation disaggregates preformed fibrils	-

EGCG, Epigallocatechin-3-gallate; TBS, tris-buffered saline.

## Abbreviations

AD	Alzheimer’s disease
Aβ	Amyloid-beta
APPswe/PS1dE9 mice	referred to as APP/PS1, double transgenic mouse model of Alzheimer’s disease over expressing amyloid precursor protein (APPswe), encoding the Swedish mutations at amino acids 595/596 and an exon-9-deleted human PS1 (PS1dE9)
TG-SwDI mice	triple transgenic mouse model of Alzheimer’s disease, express an AβPP with Aβ flanking, double Swedish mutations (Lys670→Asn/ Met671→Leu), the Dutch (Glu693→Gln), and the Iowa (Asp694→Asn) mutations (sequence numbering in the AβPP770 isoform notation)
TG2576 mice	transgenic mouse model, which express a 695-aa residue splice form of human amyloid precursor protein modified by the Swedish Familial AD double mutation K670N-M671L
TgCRND8 mice	transgenic mouse model of Alzheimer’s disease
GABA	gamma-aminobutyric acid
5XFAD mice	transgenic mice overexpress mutant human APP(695) with the Swedish (K670N, M671L), Florida (I716V), and London (V717I) Familial Alzheimer’s Disease (FAD) mutations along with human PS1 harboring two FAD mutations, M146L and L286V
APP	Amyloid Precursor Protein
PSEN1	presenilin 1
APOE	apolipoprotein E
MAPT	microtubule-associated protein tau
Trem2	triggering receptor expressed on myeloid cells 2
BACE1	Beta-Secretase 1
EGCG	Epigallocatechin-3-gallate
CTF	carboxyterminal fragments generated by α-secretase
sAPPα	generated when α-secretase cleaves APP
TNF-a	Tumor Necrosis Factor alpha
JNK	c-Jun N-terminal kinases
A11	oligomer-specific antibody
W20	oligomer-specific single chain variable fragment
TBS	tris-buffered saline
PBS	phosphate-buffered saline
DMSO	dimethyl sulfoxide
PCR	polymerase chain reaction
PMDs	protein misfolding disorders
LTP	long-term potentiation
LTD	long-term depression of excitatory synaptic transmission

## References

[1] Nie Q., Du X.G., Geng M.Y. (2011). Small molecule inhibitors of amyloid β peptide aggregation as a potential therapeutic strategy for Alzheimer’s disease. Acta Pharmacol. Sin..

[2] Weidner W.S., Barbarino P. (2018). World Alzheimer Report 2018—The State of the Art of Dementia Research: New Frontiers.

[3] Ma L., Yang C., Zheng J., Chen Y., Xiao Y., Huang K. (2020). Non-polyphenolic natural inhibitors of amyloid aggregation. Eur. J. Med. Chem..

[4] Velander P., Wu L., Henderson F., Zhang S., Bevan D.R., Xu B. (2017). Natural product-based amyloid inhibitors. Biochem. Pharmacol..

[5] Bharadwaj P.R., Dubey A.K., Masters C.L., Martins R.N., Macreadie I.G. (2008). Aβ aggregation and possible implications in Alzheimer’s disease pathogenesis. J. Cell. Mol. Med..

[6] Zhang X., Fu Z., Meng L., He M., Zhang Z. (2018). The Early Events That Initiate β-Amyloid Aggregation in Alzheimer’s Disease. Front. Aging Neurosci..

[7] Stanciu G.D., Luca A., Rusu R.N., Bild V., Chiriac S.I.B., Solcan C., Bild W., Ababei D.C. (2020). Alzheimer’s disease pharmacotherapy in relation to cholinergic system involvement. Biomolecules.

[8] Ryan P., Patel B., Makwana V., Jadhav H.R., Kiefel M., Davey A., Reekie T.A., Rudrawar S., Kassiou M. (2018). Peptides, Peptidomimetics, and Carbohydrate-Peptide Conjugates as Amyloidogenic Aggregation Inhibitors for Alzheimer’s Disease. ACS Chem. Neurosci..

[9] Lührs T., Ritter C., Adrian M., Riek-Loher D., Bohrmann B., Döbeli H., Schubert D., Riek R. (2005). 3D structure of Alzheimer’s amyloid-β(1-42) fibrils. Proc. Natl. Acad. Sci. USA.

[10] Dhouafli Z., Cuanalo-Contreras K., Hayouni E.A., Mays C.E., Soto C., Moreno-Gonzalez I. (2018). Inhibition of protein misfolding and aggregation by natural phenolic compounds. Cell. Mol. Life Sci..

[11] Yoshiike Y., Tanemura K., Murayama O., Akagi T., Murayama M., Sato S., Sun X., Tanaka N., Takashima A. (2001). New Insights on How Metals Disrupt Amyloid β-Aggregation and Their Effects on Amyloid-β Cytotoxicity. J. Biol. Chem..

[12] Yang F., Lim G.P., Begum A.N., Ubeda O.J., Simmons M.R., Ambegaokar S.S., Chen P., Kayed R., Glabe C.G., Frautschy S.A. (2005). Curcumin inhibits formation of amyloid β oligomers and fibrils, binds plaques, and reduces amyloid in vivo. J. Biol. Chem..

[13] Biancalana M., Koide S. (2010). Molecular mechanism of Thioflavin-T binding to amyloid fibrils. Biochim. Biophys. Acta - Proteins Proteomics.

[14] Maezawa I., Hong H.S., Liu R., Wu C.Y., Cheng R.H., Kung M.P., Kung H.F., Lam K.S., Oddo S., LaFerla F.M. (2008). Congo red and thioflavin-T analogs detect Aβ oligomers. J. Neurochem..

[15] Levine H. (1993). Thioflavine T interaction with synthetic Alzheimer’s disease β-amyloid peptides: Detection of amyloid aggregation in solution. Protein Sci..

[16] Groenning M. (2010). Binding mode of Thioflavin T and other molecular probes in the context of amyloid fibrils-current status. J. Chem. Biol..

[17] Liu P.-P., Xie Y., Meng X.-Y., Kang J.-S. (2019). History and progress of hypotheses and clinical trials for Alzheimer’s disease. Signal Transduct. Target. Ther..

[18] (2019). Alzheimer Association Early Signs and Symptoms of Alzheimer’s. Alzheimer’s Dement..

[19] Games D., Adams D., Alessandrini R., Barbour R., Borthelette P., Blackwell C., Carr T., Clemens J., Donaldson T., Gillespie F. (1995). Alzheimer-type neuropathology in transgenic mice overexpressing V717F β-amyloid precursor protein. Nature.

[20] Calhoun M.E., Wiederhold K.H., Abramowski D., Phinney A.L., Probst A., Sturchler-Pierrat C., Staufenbiel M., Sommer B., Jucker M. (1998). Neuron loss in APP transgenic mice [7]. Nature.

[21] Sturchler-Pierrat C., Abramowski D., Duke M., Wiederhold K.H., Mistl C., Rothacher S., Ledermann B., Bürki K., Frey P., Paganetti P.A. (1997). Two amyloid precursor protein transgenic mouse models with Alzheimer disease-like pathology. Proc. Natl. Acad. Sci. USA.

[22] Hsiao K., Chapman P., Nilsen S., Eckman C., Harigaya Y., Younkin S., Yang F., Cole G. (1996). Correlative memory deficits, Aβ elevation, and amyloid plaques in transgenic mice. Science.

[23] Holcomb L., Gordon M.N., Mcgowan E., Yu X., Benkovic S., Jantzen P., Wright K., Saad I., Mueller R., Morgan D. (1998). Accelerated Alzheimer-type phenotype in transgenic mice carrying both mutant amyloid precursor protein and presenilin 1 transgenes. Nat. Med..

[24] Richards J.G., Higgins G.A., Ouagazzal A.M., Ozmen L., Kew J.N.C., Bohrmann B., Malherbe P., Brockhaus M., Loetscher H., Czech C. (2003). PS2APP transgenic mice, coexpressing hPS2mut and hAPPswe, show age-related cognitive deficits associated with discrete brain amyloid deposition and inflammation. J. Neurosci..

[25] Moechars D., Dewachter I., Lorent K., Reversé D., Baekelandt V., Naidu A., Tesseur I., Spittaels K., Van Den Haute C., Checler F. (1999). Early phenotypic changes in transgenic mice that overexpress different mutants of amyloid precursor protein in brain. J. Biol. Chem..

[26] Jankowsky J.L., Slunt H.H., Gonzales V., Jenkins N.A., Copeland N.G., Borchelt D.R. (2004). APP processing and amyloid deposition in mice haplo-insufficient for presenilin 1. Neurobiol. Aging.

[27] Andorfer C., Kress Y., Espinoza M., De Silva R., Tucker K.L., Barde Y.A., Duff K., Davies P. (2003). Hyperphosphorylation and aggregation of tau in mice expressing normal human tau isoforms. J. Neurochem..

[28] Schindowski K., Bretteville A., Leroy K., Bégard S., Brion J.P., Hamdane M., Buée L. (2006). Alzheimer’s disease-like tau neuropathology leads to memory deficits and loss of functional synapses in a novel mutated tau transgenic mouse without any motor deficits. Am. J. Pathol..

[29] Decker J.M., Krüger L., Sydow A., Dennissen F.J., Siskova Z., Mandelkow E., Mandelkow E. (2016). The Tau/A152T mutation, a risk factor for frontotemporal-spectrum disorders, leads to NR 2B receptor-mediated excitotoxicity. EMBO Rep..

[30] Oddo S., Caccamo A., Shepherd J.D., Murphy M.P., Golde T.E., Kayed R., Metherate R., Mattson M.P., Akbari Y., LaFerla F.M. (2003). Triple-transgenic model of Alzheimer’s Disease with plaques and tangles: Intracellular Aβ and synaptic dysfunction. Neuron.

[31] Oakley H., Cole S.L., Logan S., Maus E., Shao P., Craft J., Guillozet-Bongaarts A., Ohno M., Disterhoft J., Van Eldik L. (2006). Intraneuronal β-amyloid aggregates, neurodegeneration, and neuron loss in transgenic mice with five familial Alzheimer’s disease mutations: Potential factors in amyloid plaque formation. J. Neurosci..

[32] Mullane K., Williams M. (2019). Preclinical Models of Alzheimer’s Disease: Relevance and Translational Validity. Curr. Protoc. Pharmacol..

[33] Deture M.A., Dickson D.W. (2019). The neuropathological diagnosis of Alzheimer’s disease. Mol. Neurodegener..

[34] Myers A., McGonigle P. (2019). Overview of Transgenic Mouse Models for Alzheimer’s Disease. Curr. Protoc. Neurosci..

[35] Lippi S.L.P., Smith M.L., Flinn J.M. (2018). A Novel hAPP/htau Mouse Model of Alzheimer’s Disease: Inclusion of APP With Tau Exacerbates Behavioral Deficits and Zinc Administration Heightens Tangle Pathology. Front. Aging Neurosci..

[36] Foidl B., Humpel C. (2020). Can mouse models mimic sporadic Alzheimer’s disease?. Neural Regen. Res..

[37] Ştefănescu R., Stanciu G.D., Luca A., Caba I.C., Tamba B.I., Mihai C.T. (2019). Contributions of mass spectrometry to the identification of low molecular weight molecules able to reduce the toxicity of amyloid-β peptide to cell cultures and transgenic mouse models of Alzheimer’s disease. Molecules.

[38] Liu Y., Pukala T.L., Musgrave I.F., Williams D.M., Dehle F.C., Carver J.A. (2013). Gallic acid is the major component of grape seed extract that inhibits amyloid fibril formation. Bioorganic Med. Chem. Lett..

[39] Wong D.Y.S., Musgrave I.F., Harvey B.S., Smid S.D. (2013). Açaí (Euterpe oleraceae Mart.) berry extract exerts neuroprotective effects against β-amyloid exposure in vitro. Neurosci. Lett..

[40] Porzoor A., Alford B., Hügel H.M., Grando D., Caine J., Macreadie I. (2015). Anti-amyloidogenic properties of some phenolic compounds. Biomolecules.

[41] Yu M., Chen X., Liu J., Ma Q., Zhuo Z., Chen H., Zhou L., Yang S., Zheng L., Ning C. (2019). Gallic acid disruption of Aβ1–42 aggregation rescues cognitive decline of APP/PS1 double transgenic mouse. Neurobiol. Dis..

[42] Sun J., Jiang G., Shigemori H. (2019). Inhibitory Activity on Amyloid Aggregation of Rosmarinic Acid and Its Substructures from Isodon japonicus. Nat. Prod. Commun..

[43] Hamaguchi T., Ono K., Murase A., Yamada M. (2009). Phenolic Compounds Prevent Alzheimer’s Pathology through Different Effects on the Amyloid-β Aggregation Pathway. Am. J. Pathol..

[44] Hase T., Shishido S., Yamamoto S., Yamashita R., Nukima H., Taira S. (2019). Rosmarinic acid suppresses Alzheimer ’ s disease development by reducing amyloid β aggregation by increasing monoamine secretion. Sci. Rep..

[45] Durairajan S.S.K., Yuan Q., Xie L., Chan W.S., Kum W.F., Koo I., Liu C., Song Y., Huang J.D., Klein W.L. (2008). Salvianolic acid B inhibits Aβ fibril formation and disaggregates preformed fibrils and protects against Aβ-induced cytotoxicty. Neurochem. Int..

[46] Shen L., Han B., Geng Y., Wang J., Wang Z., Wang M. (2017). Amelioration of cognitive impairments in APPswe/PS1dE9 mice is associated with metabolites alteration induced by total salvianolic acid. PLoS ONE.

[47] Akaishi T., Morimoto T., Shibao M., Watanabe S., Sakai-Kato K., Utsunomiya-Tate N., Abe K. (2008). Structural requirements for the flavonoid fisetin in inhibiting fibril formation of amyloid β protein. Neurosci. Lett..

[48] Churches Q.I., Caine J., Cavanagh K., Epa V.C., Waddington L., Tranberg C.E., Meyer A.G., Varghese J.N., Streltsov V., Duggan P.J. (2014). Naturally occurring polyphenolic inhibitors of amyloid beta aggregation. Bioorganic Med. Chem. Lett..

[49] Rezai-Zadeh K., Douglas Shytle R., Bai Y., Tian J., Hou H., Mori T., Zeng J., Obregon D., Town T., Tan J. (2009). Flavonoid-mediated presenilin-1 phosphorylation reduces Alzheimer’s disease β-amyloid production. J. Cell. Mol. Med..

[50] Sawmiller D., Li S., Shahaduzzaman M., Smith A.J., Obregon D., Giunta B., Borlongan C.V., Sanberg P.R., Tan J. (2014). Luteolin reduces Alzheimer’s disease pathologies induced by traumatic brain injury. Int. J. Mol. Sci..

[51] Pérez Corredor P., Sabogal Guáqueta A., Hormaza C., Cardona Gómez G. (2019). Preventive effect of quercetin in a triple transgenic Alzheimer’s disease mice model. Molecules.

[52] Sabogal-Guáqueta A.M., Muñoz-Manco J.I., Ramírez-Pineda J.R., Lamprea-Rodriguez M., Osorio E., Cardona-Gómez G.P. (2015). The flavonoid quercetin ameliorates Alzheimer’s disease pathology and protects cognitive and emotional function in aged triple transgenic Alzheimer’s disease model mice. Neuropharmacology.

[53] Zhang X., Hu J., Zhong L., Wang N., Yang L., Liu C.C., Li H., Wang X., Zhou Y., Zhang Y. (2016). Quercetin stabilizes apolipoprotein e and reduces brain Aβ levels in amyloid model mice. Neuropharmacology.

[54] Currais A., Prior M., Dargusch R., Armando A., Ehren J., Schubert D., Quehenberger O., Maher P. (2014). Modulation of p25 and inflammatory pathways by fisetin maintains cognitive function in Alzheimer’s disease transgenic mice. Aging Cell.

[55] Shimmyo Y., Kihara T., Akaike A., Niidome T., Sugimoto H. (2008). Multifunction of myricetin on Aβ: Neuroprotection via a conformational change of Aβ and reduction of Aβ via the interference of secretases. J. Neurosci. Res..

[56] Jia L., Zhao W., Sang J., Wang W., Wei W., Wang Y., Zhao F., Lu F., Liu F. (2019). Inhibitory Effect of a Flavonoid Dihydromyricetin against Aβ40 Amyloidogenesis and Its Associated Cytotoxicity. ACS Chem. Neurosci..

[57] Liang J., Lindemeyer A.K., Shen Y., López-Valdés H.E., Martínez-Coria H., Shao X.M., Olsen R.W. (2014). Dihydromyricetin ameliorates behavioral deficits and reverses neuropathology of transgenic mouse models of Alzheimer’s disease. Neurochem. Res..

[58] Huang Q., Zhao Q., Peng J., Yu Y., Wang C., Zou Y., Su Y., Zhu L., Wang C., Yang Y. (2019). Peptide-Polyphenol (KLVFF/EGCG) binary modulators for inhibiting aggregation and neurotoxicity of amyloid-β peptide. ACS Omega.

[59] Bieschke J., Russ J., Friedrich R.P., Ehrnhoefer D.E., Wobst H., Neugebauer K., Wanker E.E. (2010). EGCG remodels mature α-synuclein and amyloid-β fibrils and reduces cellular toxicity. Proc. Natl. Acad. Sci. USA.

[60] Rezai-Zadeh K., Shytle D., Sun N., Mori T., Hou H., Jeanniton D., Ehrhart J., Townsend K., Zeng J., Morgan D. (2005). Green tea epigallocatechin-3-gallate (EGCG) modulates amyloid precursor protein cleavage and reduces cerebral amyloidosis in Alzheimer transgenic mice. J. Neurosci..

[61] Rezai-Zadeh K., Arendash G.W., Hou H., Fernandez F., Jensen M., Runfeldt M., Shytle R.D., Tan J. (2008). Green tea epigallocatechin-3-gallate (EGCG) reduces β-amyloid mediated cognitive impairment and modulates tau pathology in Alzheimer transgenic mice. Brain Res..

[62] Li Q., Gordon M., Tan J., Morgan D. (2006). Oral administration of green tea epigallocatechin-3-gallate (EGCG) reduces amyloid beta deposition in transgenic mouse model of Alzheimer’s disease. Exp. Neurol..

[63] Jia N., Han K., Kong J.J., Zhang X.M., Sha S., Ren G.R., Cao Y.P. (2013). (-)-Epigallocatechin-3-gallate alleviates spatial memory impairment in APP/PS1 mice by restoring IRS-1 signaling defects in the hippocampus. Mol. Cell. Biochem..

[64] Adlard P.A., Perreau V.M., Pop V., Cotman C.W. (2005). Brief Communication Voluntary Exercise Decreases Amyloid Load in a Transgenic Model of Alzheimer’s Disease. J. Neurosci..

[65] Yin F., Liu J., Ji X., Wang Y., Zidichouski J., Zhang J. (2011). Silibinin: A novel inhibitor of Aβ aggregation. Neurochem. Int..

[66] Duan S., Guan X., Lin R., Liu X., Yan Y., Lin R., Zhang T., Chen X., Huang J., Sun X. (2015). Silibinin inhibits acetylcholinesterase activity and amyloid β peptide aggregation: A dual-target drug for the treatment of Alzheimer’s disease. Neurobiol. Aging.

[67] Omar S.H., Scott C.J., Hamlin A.S., Obied H.K. (2019). Olive biophenols reduces alzheimer’s pathology in SH-SY5Y cells and APPswe mice. Int. J. Mol. Sci..

[68] Grossi C., Rigacci S., Ambrosini S., Ed Dami T., Luccarini I., Traini C., Failli P., Berti A., Casamenti F., Stefani M. (2013). The Polyphenol Oleuropein Aglycone Protects TgCRND8 Mice against Aß Plaque Pathology. PLoS ONE.

[69] Wang S.W., Wang Y.J., Su Y.J., Zhou W.W., Yang S.G., Zhang R., Zhao M., Li Y.N., Zhang Z.P., Zhan D.W. (2012). Rutin inhibits β-amyloid aggregation and cytotoxicity, attenuates oxidative stress, and decreases the production of nitric oxide and proinflammatory cytokines. Neurotoxicology.

[70] Xu P.X., Wang S.W., Yu X.L., Su Y.J., Wang T., Zhou W.W., Zhang H., Wang Y.J., Liu R.T. (2014). Rutin improves spatial memory in Alzheimer’s disease transgenic mice by reducing Aβ oligomer level and attenuating oxidative stress and neuroinflammation. Behav. Brain Res..

[71] Hu B., Dai F., Fan Z., Ma G., Tang Q., Zhang X. (2015). Nanotheranostics: Congo Red/Rutin-MNPs with Enhanced Magnetic Resonance Imaging and H_2_O_2_-Responsive Therapy of Alzheimer’s Disease in APPswe/PS1dE9 Transgenic Mice. Adv. Mater..

[72] Jiang T., Zhi X.L., Zhang Y.H., Pan L.F., Zhou P. (2012). Inhibitory effect of curcumin on the Al(III)-induced Aβ42 aggregation and neurotoxicity in vitro. Biochim. Biophys. Acta - Mol. Basis Dis..

[73] Garcia-Alloza M., Borrelli L.A., Rozkalne A., Hyman B.T., Bacskai B.J. (2007). Curcumin labels amyloid pathology in vivo, disrupts existing plaques, and partially restores distorted neurites in an Alzheimer mouse model. J. Neurochem..

[74] Lim G.P., Chu T., Yang F., Beech W., Frautschy S.A., Cole G.M. (2001). The curry spice curcumin reduces oxidative damage and amyloid pathology in an Alzheimer transgenic mouse. J. Neurosci..

[75] Wang P., Su C., Li R., Wang H., Ren Y., Sun H., Yang J., Sun J., Shi J., Tian J. (2014). Mechanisms and effects of curcumin on spatial learning and memory improvement in APPswe/PS1dE9 mice. J. Neurosci. Res..

[76] Ghahghaei A., Bathaie S.Z., Bahraminejad E. (2012). Mechanisms of the effects of crocin on aggregation and deposition of ab1-40 fibrils in Alzheimer’s Disease. Int. J. Pept. Res. Ther..

[77] Ghahghaei A., Bathaie S.Z., Kheirkhah H., Bahraminejad E. (2013). The protective effect of crocin on the amyloid fibril formation of aβ42 peptide in vitro. Cell. Mol. Biol. Lett..

[78] Batarseh Y.S., Bharate S.S., Kumar V., Kumar A., Vishwakarma R.A., Bharate S.B., Kaddoumi A., States U., Road C. (2018). Crocus sativus Extract Tightens the Blood-Brain Barrier, Reduces Amyloid β Load and Related Toxicity in 5XFAD Mice. ACS Chem. Neurosci..

[79] Mei Z., Yan P., Situ B., Mou Y., Liu P. (2012). Cryptotanshinione inhibits β-amyloid aggregation and protects damage from β-amyloid in SH-SY5Y cells. Neurochem. Res..

[80] Mei Z., Zhang F., Tao L., Zheng W., Cao Y., Wang Z., Tang S., Le K., Chen S., Pi R. (2009). Cryptotanshinone, a compound from Salvia miltiorrhiza modulates amyloid precursor protein metabolism and attenuates β-amyloid deposition through upregulating α-secretase in vivo and in vitro. Neurosci. Lett..

[81] Kai T., Zhang L., Wang X., Jing A., Zhao B., Yu X., Zheng J., Zhou F. (2015). Tabersonine Inhibits Amyloid Fibril Formation and Cytotoxicity of Aβ(1-42). ACS Chem. Neurosci..

[82] Xu J., Wei K., Zhang G., Lei L., Yang D., Wang W., Han Q., Xia Y., Bi Y., Yang M. (2018). Ethnopharmacology, phytochemistry, and pharmacology of Chinese Salvia species: A review. J. Ethnopharmacol..

[83] Khan H., Ullah H., Aschner M., Cheang W.S., Akkol E.K. (2019). Neuroprotective Effects of Quercetin in Alzheimer’s Disease. Biomolecules.

[84] Perret D., Luo Z.D. (2009). Targeting voltage-gated calcium channels for neuropathic pain management. Neurotherapeutics.

[85] Ma Q., Cai S., Jia Y., Sun X., Yi J., Du J. (2020). Effects of hot-water extract from vine tea (Ampelopsis grossedentata) on acrylamide formation, quality and consumer acceptability of bread. Foods.

[86] Adler B.L., Yarchoan M., Hwang H.M., Louneva N., Blair J.A., Palm R., Smith M.A., Lee H.G., Arnold S.E., Casadesus G. (2014). Neuroprotective effects of the amylin analogue pramlintide on Alzheimer’s disease pathogenesis and cognition. Neurobiol. Aging.

[87] Hussain H., Hussain J., Al-Harrasi A., Green I.R. (2012). Chemistry and biology of the genus Voacanga. Pharm. Biol..

[88] Ștefănescu R., Lupu L., Manea M., Iacob R.E., Przybylski M. (2018). Molecular characterization of the β-amyloid(4-10) epitope of plaque specific Aβ antibodies by affinity-mass spectrometry using alanine site mutation. J. Pept. Sci..

[89] Bazoti F.N., Bergquist J., Markides K., Tsarbopoulos A. (2008). Localization of the noncovalent binding site between amyloid-beta-peptide and oleuropein using electrospray ionization FT-ICR mass spectrometry. J. Am. Soc. Mass Spectrom..

[90] Bazoti F.N., Bergquist J., Markides K.E., Tsarbopoulos A. (2006). Noncovalent interaction between amyloid-beta-peptide (1-40) and oleuropein studied by electrospray ionization mass spectrometry. J. Am. Soc. Mass Spectrom..

[91] Meng F., Marek P., Potter K.J., Verchere C.B., Raleigh D.P. (2008). Rifampicin does not prevent amyloid fibril formation by human islet amyloid polypeptide but does inhibit fibril thioflavin-T interactions: Implications for mechanistic studies of β-cell death. Biochemistry.

[92] Kroes-Nijboer A., Lubbersen Y.S., Venema P., van der Linden E. (2009). Thioflavin T fluorescence assay for β-lactoglobulin fibrils hindered by DAPH. J. Struct. Biol..

[93] Hudson S.A., Ecroyd H., Kee T.W., Carver J.A. (2009). The thioflavin T fluorescence assay for amyloid fibril detection can be biased by the presence of exogenous compounds. FEBS J..

[94] Molino S., Dossena M., Buonocore D., Ferrari F., Venturini L., Ricevuti G., Verri M. (2016). Polyphenols in dementia: From molecular basis to clinical trials. Life Sci..

[95] Mandel S., Amit T., Reznichenko L., Weinreb O., Youdim M.B.H. (2006). Green tea catechins as brain-permeable, natural iron chelators-antioxidants for the treatment of neurodegenerative disorders. Mol. Nutr. Food Res..

[96] Zhang X., Wu M., Lu F., Luo N., He Z.P., Yang H. (2014). Involvement of α7 nAChR signaling cascade in epigallocatechin gallate suppression of β-Amyloid-Induced apoptotic cortical neuronal insults. Mol. Neurobiol..

[97] De Oliveira M.R., Nabavi S.F., Daglia M., Rastrelli L., Nabavi S.M. (2016). Epigallocatechin gallate and mitochondria - A story of life and death. Pharmacol. Res..

[98] Lee J.H., Moon J.H., Kim S.W., Jeong J.K., Nazim U.M.D., Lee Y.J., Seol J.W., Park S.Y. (2015). EGCG-mediated autophagy flux has a neuroprotection effect via a class III histone deacetylase in primary neuron cells. Oncotarget.

[99] Pogačnik L., Pirc K., Palmela I., Skrt M., Kwang K.S., Brites D., Brito M.A., Ulrih N.P., Silva R.F.M. (2016). Potential for brain accessibility and analysis of stability of selected flavonoids in relation to neuroprotection in vitro. Brain Res..

[100] Noble W. (2010). Challenges in neurodegeneration research. Front. Psychiatry.

[101] Serban D., Anton E., Chirita R., Bild V., Ciobica A., Alexinschi O., Arcan O., Popescu R., Paduraru L., Timofte D. (2015). Current aspects of the interactions between dementia, the brain renin-angiotensin system and oxidative stress. Arch. Biol. Sci..

[102] Patel S.S., Acharya A., Ray R.S., Agrawal R., Raghuwanshi R., Jain P. (2020). Cellular and molecular mechanisms of curcumin in prevention and treatment of disease. Crit. Rev. Food Sci. Nutr..

[103] Ringman J.M., Frautschy S.A., Teng E., Begum A.N., Bardens J., Beigi M., Gylys K.H., Badmaev V., Heath D.D., Apostolova L.G. (2012). Oral curcumin for Alzheimer’s disease: Tolerability and efficacy in a 24-week randomized, double blind, placebo-controlled study. Alzheimer’s Res. Ther..

[104] Baum L., Lam C.W.K., Cheung S.K.K., Kwok T., Lui V., Tsoh J., Lam L., Leung V., Hui E., Ng C. (2008). Six-month randomized, placebo-controlled, double-blind, pilot clinical trial of curcumin in patients with Alzheimer disease [7]. J. Clin. Psychopharmacol..

[105] Barril X. (2012). Druggability predictions: Methods, limitations, and applications. Wiley Interdiscip. Rev. Comput. Mol. Sci..

[106] Hopkins A.L., Groom C.R. (2002). The druggable genome. Nat. Rev. Drug Discov..

